# Prophylactic Knee Bracing in Offensive Linemen of the National Football League: A Retrospective Analysis of Usage Trends, Player Performance, and Major Knee Injury

**DOI:** 10.1177/23259671231191767

**Published:** 2023-08-25

**Authors:** Devon R. Ackerman, Anna M. Ptasinski, Travis Edmond, Mark L. Dunleavy, Robert A. Gallo

**Affiliations:** † College of Medicine, The Pennsylvania State University, Hershey, Pennsylvania, USA.; *Investigation performed at the College of Medicine, The Pennsylvania State University, Hershey, Pennsylvania, USA*

**Keywords:** football (American), functional knee brace, knee injury, National Football League, offensive lineman, prophylactic knee brace

## Abstract

**Background:**

Offensive linemen in American football are prone to high-energy valgus forces to the knee, leading to associated injuries. Some offensive linemen in the National Football League (NFL) wear prophylactic knee braces (PKB) to prevent ligamentous injury.

**Purpose/Hypothesis:**

This purpose of the study was to compare injury rates and performance between NFL offensive linemen who wear PKB and those who do not. It was hypothesized that brace wear would be associated with fewer major knee injuries and no difference in gameplay performance.

**Study Design:**

Cohort study; Level of evidence, 3.

**Methods:**

For the 2014 through 2020 NFL seasons, offensive linemen with at least 200 game snaps per regular season were identified. Players were grouped by PKB status (bracers vs nonbracers) based on visualization of bilateral, dual-hinged metal knee braces as part of gameday uniforms on publicly available imaging databases and/or game videos. Major knee injuries, defined as those requiring the missing of games, were identified using publicly available data. Performance was assessed with Pro Football Focus grades for each season. Rates of major knee injury were compared between groups with the 2-sample *Z* test for proportions, and performance grades were compared with the unpaired *t* test.

**Results:**

For the cumulative study period, bracers demonstrated a significantly lower rate of major knee injuries than nonbracers (0.013 vs 0.049 injuries per player, respectively; *P* = .04). Isolated MCL injury was the most common injury for nonbracers. There was no group difference in performance for the cumulative study period or during most individual seasons. Yearly prevalence of PKB usage declined steadily from 16.3% in 2014 to 5.6% in 2020. A subgroup analysis of rookie players demonstrated an overall downtrend in usage during the study period as well.

**Conclusion:**

Results indicated that knee brace prophylaxis by NFL offensive linemen was associated with a reduced risk of major knee injury without a significant difference in performance when compared with nonbracers. Despite this, the prevalence of PKB declined over the study period.

Injury to the exposed knee within American football is a well-known phenomenon, particularly at the higher levels of play. An epidemiologic study reviewing 16 years of collegiate football seasons found the knee to be the most common site of injury and accounts for 18% of injuries.^11^ In the same study, knee injuries were also the most common “severe injury,” defined as those associated with more than 10 days of time loss from sport.^11^ At the 2005 National Football League (NFL) combine, almost 54% of elite college participants reported a history of knee injury.^9^ Moreover, a retrospective analysis of 3 recent NFL seasons found the knee to be the most injured lower extremity region among all players,^18^ making up approximately 30% of injuries located within the lower extremity. In particular, medial collateral ligament (MCL) tear was highlighted as among the most common lower extremity injuries in that study.^18^

The use of a rigid, form-fitting knee brace to prevent or lessen ligamentous injury has been implemented for over 40 years and began in the late 1970s with the advent of the laterally hinged, metal “stabler” knee brace.^4,30^ This brace was proclaimed to not only prevent reinjury of the MCL during return-to-play but also decrease the risk of ligamentous injury from a lateral force in an otherwise healthy knee.^4,20,27,30^ Brace designs have since evolved, and the fundamental characteristics include either a metal-based, unilateral-hinged bar or, more commonly, upright bilateral-hinged bars, with proximal and distal shells or straps for securement.^23,27^ The American Academy of Orthopaedic Surgeons (AAOS) recognizes 2 main types of braces: prophylactic (braces intended to prevent or reduce the severity of knee injury) and functional (braces designed to reinforce natural stiffness of an unstable knee against joint stresses).^12,23,27,30^ Given the risk of high-energy valgus forces during blocking for the offensive lineman position, prophylactic knee bracing (PKB) has gained traction at this position within the highest levels of American football.^7,23^

Despite the ongoing use of PKB within the elite levels of American football, the supporting evidence for efficacy remains inconclusive; 3 overlapping systemic reviews conclude lacking evidence to fully endorse PKB as an intervention to prevent or decrease the severity of ligamentous injury in all football players.^25,29,33^ These findings are congruent with the most recent position statement by the AAOS on PKB,^12,29^ which cites some evidence for MCL protection within select football positions but ultimately concludes that there is insufficient evidence for PKB recommendation in football players generally.^1,2,3,32^ Another unsettled consideration of PKB is its influence on performance. The concept of performance hindrance is a real concern, albeit with conflicting evidence,^3,8,15,20,22,27^ while the emerging concepts of brace wear accommodation and fixed restrictive thresholds in stronger and/or larger athletes suggest negligible influence of PKB on performance.^3,19,26,27,31^

There is a scarcity of published studies focusing on PKB at the professional level. Although PKB use by NFL offensive linemen is currently practiced, the benefit in terms of injury reduction versus interference with player performance has yet to be elucidated.

The purpose of this study was to contribute a framing investigation of PKB usage and relevant outcomes within the professional level of American football. Specifically, we aimed to quantify current PKB usage trends so as to assess potential associations with player game performance and knee injury rate. Given previous evidence of knee injury reduction, primarily of the MCL, in at-risk positions such as offensive linemen,^2,9,20^ combined with the idea that offensive linemen may experience brace wear accommodation and overcome the brace’s fixed restrictive force threshold,^3,19,26,27,31^ we hypothesized that NFL offensive linemen with PKB will demonstrate a lower knee injury rate without a difference in gameplay performance.

## Methods

### Participant Selection

Data from the 2014 to 2020 NFL seasons were analyzed. NFL offensive linemen who participated in at least 200 offensive plays at a primary blocking position (tackle, guard, center) in regular season games were identified from Pro Football Focus (PFF), an online NFL player performance analysis company that provides individual player regular season blocking snap count data as part of their standardized player performance evaluation. The tight-end position was not considered a primary blocking position, and plays recorded at the tight-end position did not count toward the 200 minimum play requirements.

### Bracing Designation

Players satisfying the minimal 200 snap count for each designated season were grouped by bracing status into a PKB group or no PKB (nPKB) group. To assess bracing usage, the gameday outfit for each player was inspected visually using online public resources, such as Getty Images and Associated Press imaging. For players in whom visualization of images from the databases did not provide adequate or definitive information regarding their bracing status, still images isolated from video footage of the player from games played in that season were analyzed. Video footage was obtained from an online streaming service, NFL Game Pass.

Using both listed image databases, bracing status designation for each player was made through a differential visualization process. An initial screening for brace status was performed on all players. The initial screening consisted of viewing images from 3 regular season games: 1 game within the month of September, 1 game within the timeframe of October through November, and a third game within the month of December. These games corresponded to the beginning, middle, and end of the regular season for each season, respectively. If the bracing status for all 3 games was congruent for a given player, that status was considered final without further review. However, if there was a discrepancy between the bracing designation for any of the 3 initial games reviewed, all regular season games for the given player were reviewed. The bracing status for each game was individually recorded.

Criteria used to define brace usage based on image and/or video inspection were as follows:

1. Presence of bilateral knee braces;
2. Braces included dual medial and lateral hinge mobility with metal support frame consistent with a prophylactic or functional type knee brace;
3. Player satisfied both criteria 1 and 2 for >75% of the games played during the designated season.

Given that most NFL uniforms included knee-height socks potentially covering part of a brace, detail was given to visualizing brace silhouette comprised of a distinct contour, size, rigidity, and strap and hinge design impressed within the covering sock and/or pant material about the upper leg, knee, and distal thigh regions when direct visualization was not possible. All images were studied by the same primary image reviewer (**D.A.)**, and any questionable bracing status designations were decided by deliberation among the authors. Players not wearing any form of knee support, wearing only 1 brace, wearing bilateral nonmetal support devices or sleeves, or wearing braces for <75% of total games played in were placed in the nPKB group. Identification of brand and specific model of prophylactic/functional knee brace was not included in the analysis.

### Performance Quantification

Player performance was assessed quantitively via standardized performance data from the publicly available PFF database. Performance metrics associated with the skillset of offensive linemen were identified from the PFF database. These metrics included overall offense, run block, and pass block. The PFF grading system is based on a composite analysis of player performance at the singular play level rather than traditional statistics of offensive linemen (“pancakes,” “road-grade,” sacks allowed, etc). Trained PFF analysts assign a 0.5 incremental plus-minus (-2 to +2) score for each play based on expected versus actual outcomes by the individual player. Outcome score values are standardized using curated rubrics that define expected against possible outcomes for the various situations of the offensive lineman position. Scores from multiple independent PFF analysts are reviewed and arbitrated into a final score by a senior analyst. Finalized plus-minus scores are then converted into the 0 to 100 scale at the game and season level using proprietary formulas accounting for player consistency and situational influence. Season-level grades for each of the identified performance metrics were obtained for all included players. A more in-depth explanation of the grading system may be found on the PFF website (https://www.pff.com).^17^

PFF performance grades were used to calculate average season offense, pass, and run block scores for the PKB and nPKB groups. In addition, the difference in overall offense score between 2 consecutive seasons was calculated for players having performed in at least 2 consecutive seasons within the study period. This represented a yearly change score, which was calculated in an attempt to better control for individual skill variation and age-related performance decline.^13^ For example, a player receiving the same season overall offense score in 2 consecutive seasons would receive a yearly change score of “0”. The average yearly change in overall offense grade was obtained for the PKB and nPKB groups.

### Determination of Major Knee Injury

A major knee injury was defined as one requiring missing games. These injuries included but were not limited to MCL injury, anterior cruciate ligament (ACL) injury, patellar dislocation, patellar or quadriceps tendon tears, and/or meniscal pathology. Major knee injuries sustained by NFL offensive linemen meeting inclusion criteria during the years 2014 to 2020 were identified using publicly available data. Sources included published injury reports, news articles, and team statements. Injuries were verified through correlation with multiple reputable sources. For each identified major knee injury, the date and type of injury were determined. The total number of major knee injuries within the PKB and nPKB cohorts was determined for the cumulative study period. Injury rates were calculated by dividing the number of injuries by the total number of players in each cohort.

### Statistical Analysis

An unpaired *t* test analysis was used to compare mean PFF grades between the PKB and nPKB cohorts for each season as well as the cumulative study period. An unpaired *t* test analysis was also used to compare mean yearly change in PFF grades between the study groups for the cumulative study period. A post hoc sample size analysis of the cumulative performance scores for the PKB and nPKB groups was performed using an online statistical calculator to gauge adequacy of cohort sizes.^14^ A 2-sample *Z* proportion test was utilized to compare injury rates between the PKB and nPKB groups. The threshold for statistical significance was set at *P* < .05.

## Results

**Figure 1.**
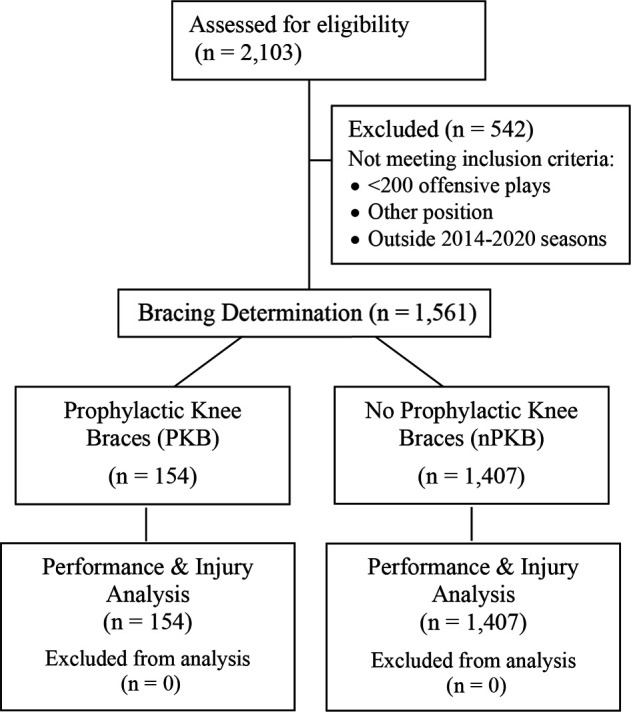
Modified CONSORT participant flowchart. CONSORT, Consolidated Standards of Reporting Trials.

A total of 1561 player entries within the 7-season study period met inclusion criteria and were subsequently analyzed for PKB usage status, major knee injury occurrence, and player performance as characterized by PFF scores in overall offensive season, pass block, and run block grades (Figure 1). There were 154 players in the PKB group and 1407 players in the nPKB group.

A post hoc sample-size analysis using the observed parameters from the study and a pre-established power of 80% and alpha value of 0.05 yielded calculated minimal cohort sizes of 137 for the PKB group (154 observed) and 1497 for the nPKB group (1407 observed) with a total participant size of 1634 (1561 observed). The observed parameters used included a baseline mean 66, standard deviation 11, minimal detectable effect (MDE) 1.0, and sampling ratio 10.945. When the MDE was increased to 1.1, the calculated minimal cohort sizes were 113 for the PKB group (154 observed) and 1237 for the nPKB group (1407 observed), with a total participant size of 1350 (1561 observed).

**Figure 2.**
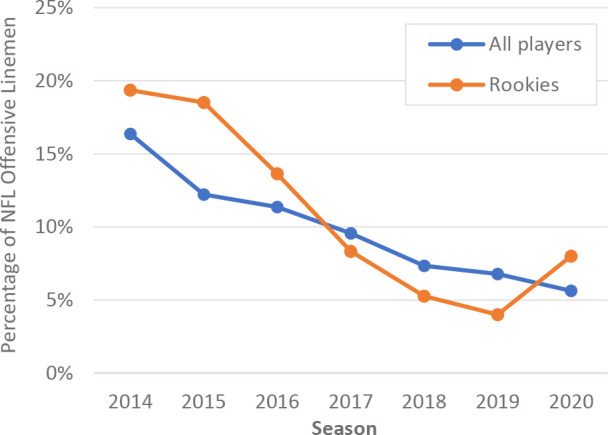
Prevalence of prophylactic knee bracing prevalence among NFL offensive linemen by year, 2014 to 2020. NFL, National Football League.

Each season yielded a similar number of players eligible for analysis, ranging from 218 to 231 players. The yearly prevalence of bracing use decreased every year throughout the study period starting with 16.3% of players in 2014 to 5.6% usage in 2020 (Figure 2). Within the total player cohort, 161 first-year players (rookies) were identified. The prevalence of bracing use within the rookie subgroup demonstrated an overall decline from 19.4% in 2014 to 4.0% and 8.0% in 2019 and 2020, respectively (Figure 2). The overall prevalence of brace usage for the total study period was 9.9% for the total player group and 11.8% for the rookie subgroup.

Overall season grades by year and for the cumulative study period are summarized in Table 1. There was no difference between the average PFF season overall, pass block, and run block score between the PKB and nPKB groups for the overall study period of 2014 to 2020 or for most individual seasons. Notably, the 2015 season did yield significant differences among performance scores for 2 out of 3 metrics in favor of the nPKB group: average overall offensive grades were 67.9 versus 63.1 (*P* = .03) and average season pass block grades were 69.2 versus 61.9 (*P* = .01). Run blocking was also higher for nonbracers but nonsignificant: 65.4 and 62.8 (*P* = .16). Performance metric scores for all other seasons had no difference. The average yearly change in overall offensive PFF grade was 0.2 ± 10.9 for the PKB group compared with -0.8 ± 9.4 for the nPKB group (*P* = .44).

**Table 1:** Performance in NFL Offensive Linemen According to Study Group*^a^*

	PFF Overall Grade, Mean ± SD		
Year	PKB Group	nPKB Group	*P*
2014 (n = 220)	66.1 ± 9.8 (n = 36)	69.4 ± 10.5 (n = 184)	.07
2015 (n = 221)	63.1 ± 9.9 (n = 27)	67.9 ± 10.6 (n = 194)	**.03**
2016 (n = 220)	69.1 ± 11.7 (n = 25)	69.7 ± 10.5 (n = 195)	.80
2017 (n = 230)	66.4 ± 11.5 (n = 22)	64.5 ± 10.9 (n = 208)	.46
2018 (n = 218)	68.6 ± 9.9 (n = 16)	64.8 ± 10.7 (n = 202)	.16
2019 (n = 221)	61.6 ± 12.3 (n = 15)	63.9 ± 10.8 (n = 206)	.49
2020 (n = 231)	69.1 ± 12.2 (n = 13)	64.9 ± 11.6 (n = 218)	.24
Cumulative (n = 1561)	66.2 ± 11.0 (n = 154)	66.3 ± 11.0 (n = 1407)	.85

*^a^* Boldface *P* value indicates statistically significant difference between groups (P < .05). NFL, National Football League; PFF, Pro Football Focus; nPKB, no prophylactic knee braces; PKB, prophylactic knee braces.

**Figure 3.**
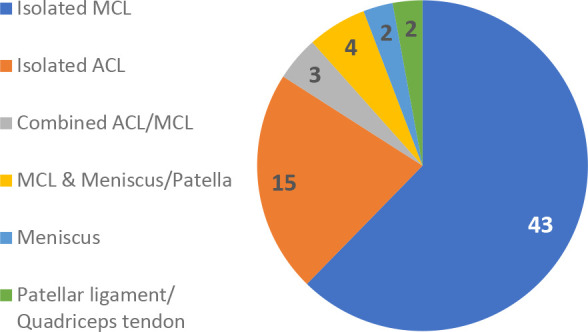
Types of knee injury among offensive linemen in the nPKB group. ACL, anterior cruciate ligament; MCL, medial collateral ligament; nPKB, no prophylactic knee braces.

Major knee injury data analysis revealed a greater number of injuries (N = 69) among the nPKB group compared with the PKB group (N = 2) during the 7 consecutive season study periods. Given totals of 1407 players in the nPKB group and 154 players in the PKB group, this equated to significantly different injury rates of 0.049 and 0.013 injuries per player for the nPKB and PKB groups, respectively (*P* = .04). Specific major knee injuries for the nPKB group included 43 isolated MCL injuries, 15 isolated ACL tears, 3 combined ligamentous injuries involving ACL and MCL pathology, 4 MCL injuries with concomitant meniscus tear or patella dislocation, 2 isolated meniscal tears, and 2 isolated patellar ligament or quadricep tendon tears (Figure 3). The injuries of the PKB group included 1 MCL sprain and 1 isolated ACL tear.

## Discussion

For the overall study period, a significant decrease in the rate of major knee injury in bracers versus nonbracers was observed. This finding aligns with the most recent position statement by the AAOS on the practice of PKB,^12,29^ which asserts that knee brace prophylaxis may have a role in MCL protection for select football positions. The AAOS position was based on the results of a biomechanical study which demonstrated a 20% to 30% greater resistance by off-the-shelf prophylactic knee braces (PKB) when applied to a fully extended surrogate knee model in response to a significant lateral force with magnitude sufficient to open the medial joint line.^3,12,20,27^ Also implicated were the results of 2 major clinical studies. First, a prospective randomized study on PKB revealed a statistically significant reduction of MCL injury in intramural tackle football players wearing prophylactic braces^12,20,27,32^; second, a study of Big Ten Conference football players showed a consistent, although statistically insignificant, reduction of MCL injury among braced players in high-risk position groups,^1,2^ including offensive linemen.^20^ Another source of continued support for PKB, perpetuated within coaching and player communities, is anecdotal stories of player knee collisions resulting in visibly bent or damaged knee braces without actual injury to the player’s joint.^8,24^

Regarding injury type, MCL pathology was the most common type observed in the combined cohort. This is consistent with previous literature describing the risk of knee injury to offensive linemen, specifically from valgus stress. Albright et al^2,20^ found the offensive lineman position group to have more than double the number of MCL injuries than the next position group within their Big Ten Conference bracing study, and another study also demonstrated a linkage between offensive linemen and MCL injury.^9^ Offensive linemen are vulnerable because their main responsibility is to block opposing defenders within the confined space of the line-of-scrimmage. This places them near adjacent offensive linemen and defenders, creating opportunities for multiple injury mechanisms. Offensive linemen are subject to unforeseen acute valgus force from other players falling or “rolling up” onto the lateral aspect of the player’s knee. In addition, offensive linemen are subject to certain “reverse-blocking” techniques, termed “cut” or “chop-blocks,” whereby defensive players will undercut offensive linemen by throwing their body toward the offensive lineman at the level of the knee, either head-on or obliquely, resulting in further acute stress on the knee joint.^9^

Performance hindrance due to knee brace wear represents a legitimate concern against prophylactic use. Previous kinematic, functional, and sports-related performance testing studies suggest braces may hinder athletic performance by decreasing straight-ahead sprinting speed and agility, decreasing muscular strength and power, increasing muscular relaxation pressure and energy expenditure, and altering neuromuscular control.^3,8,15,22,27^ Researchers postulate that braces exert these detrimental effects via early fatigue to the wearer owing to added brace weight, brace slippage, external force on the joint, and tissue compression from brace straps and/or brace fixation systems.^3,15,27^ However, other researchers have suggested that none or negligible differences in performance exist.^3,20,27^ In our study, no difference in player performance, as measured by standardized season performance scoring, was observed between bracers versus nonbracers for the cumulative study period. Moreover, yearly change scores, which served to control for age-related skill decline and individual player skill variation, also remained similar regardless of PKB designation. Because of the relative novelty of the PFF scoring system for performance evaluation, there is no consensus on the MDE in PFF scoring system. Post hoc sample size calculations corresponding to a power of 80% and alpha value of 0.05 determined that our sample size was adequately powered to detect an MDE of 1.1.

Although the cumulative study analysis demonstrated no difference in performance, the individual 2015 season yielded notably lower overall and pass blocking scores for bracers. On the surface, this may offer support for the argument of performance hindrance by brace wear. However, we believe the results from the 2015 season to be from chance given the inconsistent trending in performance scores across the total study period. In subsequent seasons, there are times that the overall season grade and pass block scores conversely favor bracers. To our knowledge, no change to the types of braces used during the 2015 season occurred, effectively ruling out intrinsic brace design as an influence on performance.^15^ Furthermore, if owing to the brace, we would expect hindrance to be more profound in the run blocking metric as opposed to pass blocking. Run blocking entails using one’s lower body to move a defender backward while pass blocking is more reliant on leverage and maintaining position. Given the greater work and energy expenditure, run blocking would be more prone to early fatigue with the added brace wear. However, this was not the case for the 2015 season where run blocking scores were similar.

The findings from our study may highlight an important aspect of the brace-performance question: the brace wearer. Sforzo et al^31^ alluded to this in their 1989 study, which examined the performance effects of bracing on asymptomatic college athletes. They found that the overall performance of female lacrosse players, as measured by quadriceps peak torque, rise time, time to fatigue, anaerobic power, and blood lactate accumulation, was inhibited with the use of a prophylactic brace, whereas the performance of male football players assessed across the same parameters was not significantly affected. This observation leads to the theory of a fixed restrictive force threshold imposed by knee braces. Male athletes, particularly offensive linemen who represent the heaviest and strongest position in American football, generally possess greater lower extremity muscle mass and strength. Therefore, they may be able to overcome the fixed restrictive forces of braces more easily. The theory was promulgated in a more recent randomized control trial of young, healthy male collegiate athletes performing various power and agility-based testing in the presence or absence of neoprene sleeves and PKB in crossover design fashion.^19^ They found no statistically significant difference in mean muscular strength and power despite minimal reductions in peak torque during isokinetic flexion and extension testing (3.2% and 0.4%, respectively) with brace wear. The researchers speculated that the participants were able to generate a mean peak torque large enough to compensate for the imposed joint restriction.

Related yet potentially distinct is the emerging concept of brace accommodation by which athletes display fewer or no effects of donning a brace over time due to restrictive force adaptation.^3,26,27^ The exact mechanisms of adaptation and the latency period threshold have yet to be elucidated, but researchers suggest this process circumvents ongoing neuromuscular disruption of the knee joint with brace use.^26,27^

PKB prevalence for the overall cohort demonstrated a yearly drop in usage for each consecutive season studied. First-year players within the league demonstrated an overall downtrend in PKB usage throughout the study period despite a modest uptick in 2020. This observation in first-year players may partially explain the overall decreased prevalence rate within the league at large. The decreased bracing rate in NFL offensive linemen is in stark contrast to other levels within the realm of elite American football. Bracing prevalence in offensive linemen at the Division I FBS collegiate level has increased since the 1990s and currently remain near 100% for games.^7^ Reasons behind the decline in PKB usage at the professional level are likely multifactorial and inherently associated with the overall governance of player rules by the NFL.

Health and safety standards are implicated in the collective bargaining agreement between the league’s players and ownership, which prevents a widespread knee bracing mandate. Team-wide mandates exist but do not account for all players with PKB use within our study, suggesting that bracing decisions are mainly player specific.^6,7,24,28^ In addition to perceived performance hindrance, which is of notable concern for professional athlete compensation and career,^27^ other negative perceptions of knee braces exist and may factor into player decisions for PKB. Knee braces can be uncomfortable due to their restrictive or cumbersome feel, irritation to skin, or have an unpleasant odor that accompanies ongoing use. Other player-specific factors may include, but are not limited to, previous bracing experiences, previous injuries, and teammate and medical staff influence. Future investigation in the form of a league-wide survey of training staff, coaches, and players inquiring about the decision for PKB would be helpful to better understand the decreasing prevalence.

Given this is a first-in-kind study, precedent inclusion criteria for players were unavailable. The choice of a 200-snap minimum requirement per season was selected to capture players that were starters or significant reserves in their respective offensive lineman position. This snap count equated to about 4 full games, or a quarter of the season, considering the average total offensive plays per game is around 50 plays,^21^ and offensive linemen typically play the whole game without substitution. Including only starters and contributing reserve players allowed us to better control baseline player performance and PKB decisions. Consequently, the generalizability of this study is only to starters, not the whole NFL offensive linemen cohort.

Another important component of our study was the decision to focus on the in-game setting. Researchers have discovered that knee injuries are much more common during game settings, as opposed to practice, for the NFL and college levels.^2,11,16,18^ This has led to the speculation that the intensity of session is a major determinant of knee injury occurrence. Albright et al^1^ found the MCL sprain rate of football players to be 6 to 12 times greater in games compared with the rate associated with practices and virtually nonexistent in noncontact situations; they postulated this difference as arising from the disparity in physical intensity and degree of contact.^1^ Given the NFL has greatly restricted the degree of full-contact within practices,^10^ the most relevant area of focus for knee injury risk and subsequent mitigations would be within games. As such, we believe the omission of knee exposure and potential injury incidence contribution from the practice setting is inconsequential in terms of the overall injury rate per player and evaluation of PKB utility. Nonetheless, the exclusion of the practice setting from our study potentially missed a theoretical cohort of players preferentially using prophylactic braces in practice alone. Future investigation is needed to (1) clarify the current application of PKB within the practice setting and (2) determine the potential role of PKB use in practices from an injury alleviation perspective.

### Limitations

Our study is not without limitations. Inherent to the retrospective observational nature of our study design is the risk for selection bias. The decision for PKB was largely player-dependent and determined by factors discussed previously. A large discrepancy between cohort size in favor of nonbracers was observed. Although all players included in the study were elite offensive linemen designated as starters or contributing reserves with comparable skill, the mean baseline player skill in each group may have been unequal owing to skewed PKB preferences. We attempted to control for baseline player skill through the determination of yearly change scores. However, potential differences in performance from brace wear may have been masked in our analysis of average PFF scores. Moreover, baseline knee injury risk may have been different among the 2 cohorts owing to discrepancies in various factors, namely previous knee injury. Past medical history for each player was not readily available, so we were unable to control for this potential confounder. However, we speculate that if there was a difference in baseline knee injury risk, it was greater in bracers who opted for extra knee stabilization and protection.

Our methods lacked the specificity to determine explicit brace brand and design. As a result, we did not control for brace type in this study. Rather, all braces, once deemed functional or prophylactic metallic braces, were considered as a single conglomerate. A previous study from 2000 found that some off-the-shelf braces were associated with decreased speed, agility, and variable migration about the knee joint during use.^15^ Although most of the types of braces used in that study have been updated or phased out for newer brace models, heterogeneity of brace type may have affected performance outcomes in our study. Moreover, current knowledge about the assortment of braces used at the NFL level is lacking. Supplemental investigation of current brace options and their respective functionality profiles is needed in the ongoing exploration of PKB by NFL offensive linemen.

Publicly available data were used for bracing status designation and knee injury data. Our methodology for inspection of high-resolution, player images from image databases and gameday footage stills allowed for a practical and efficient evaluation of PKB usage across the professional league in its entirety. Although relating a binary observation (presence or absence of braces), this image review process is novel and unvetted. It also assumes that bilateral brace wear by a player is for prophylactic use. Although this is likely the case, it was not possible to confirm whether the player was wearing bilateral braces to prevent future injury or stabilize a current one. Further, knee injury data were obtained from publicly available sources as opposed to league medical records. Consequently, the exact details, including the mechanism and grade of ligamentous injury, were not always known. Nonetheless, this approach to sports injury identification has been used in previously published work.^5,34,35^ The PFF grading system was chosen because it afforded a standardized and quantifiable approach to compare relevant gametime performance metrics of the offensive lineman position. PFF grading as a proxy for NFL player performance has been used in a previously published study as well as in mainstream media.^35^

Despite these limitations, our data demonstrated that (1) the prevalence of PKB by NFL offensive linemen is decreasing despite showing (2) PKB use is associated with significantly fewer major knee injuries among NFL offensive linemen, and (3) PKB use is not associated with a difference in offensive linemen gameplay performance. To our knowledge, this is the first study to examine PKB use by NFL offensive linemen with regard to injury rate and performance. Further, this is the first to characterize bracing influence on performance using a standardized scoring system of actual gameplay as opposed to biomechanical or functional testing. Supplemental investigations of player and staff surveys will help to confirm reasons for decreasing PKB prevalence, understand the applicability of PKB to the practice setting, and evaluate the common brace types currently employed by NFL offensive linemen.

## Conclusion

Although the prevalence of prophylactic bracing by NFL offensive linemen decreased yearly from the 2014 to 2020 seasons, prophylactic braces were associated with fewermajor knee injuries without a difference in performance. While these findings are encouraging, further investigations are needed before a stance may be taken on the medical utility of PKB in this select cohort of football players. It is our hope that this study contributes foundational impetus for future studies to aid in shaping PKB practices at the professional level.
